# EFFECTS OF COVID-19 PANDEMIC ON OLDER WORKERS’ LABOR MARKET AND RETIREMENT DECISIONS

**DOI:** 10.1093/geroni/igad104.1150

**Published:** 2023-12-21

**Authors:** Siavash Radpour

**Affiliations:** Stockton University, Galloway, New Jersey, United States

## Abstract

Several studies have documented the increase in retirement since the onset of the Covid-19 pandemic. I contribute to this literature by answering two key questions that show the pandemic excess retirement is caused primarily by demand-side factors: first, using the monthly Current Population Survey (CPS) data on employment and retirement of older workers and CPS’s panel structure, I explore if older workers left their jobs and the labor force voluntarily as a part of “the great resignation”. I find that very few workers quit their jobs voluntarily, and most retirements were preceded by involuntary job loss and unemployment. Second, using the Health and Retirement Study (HRS) with its rich data on wealth, employment, and the special Covid-19 related questions included in 2020 HRS (Wave 15), I examine if older workers who experienced job loss during the pandemic retired because they were financially prepared for retirement. I find that among the small group of older workers who quit their jobs because of the pandemic, the majority did not have adequate retirement assets and their decision is more likely to be based on their health status and vulnerability during the pandemic.

